# Gasdermin E: a missing link in muscle regeneration

**DOI:** 10.1172/JCI205442

**Published:** 2026-04-15

**Authors:** Swathy Krishna, Jill A. Rafael-Fortney

**Affiliations:** Department of Physiology and Cell Biology, College of Medicine, The Ohio State University, Columbus, Ohio, USA.

## Abstract

Skeletal muscle has the impressive capacity to completely regenerate even after relatively severe injuries in young individuals, but this process is dysregulated in multiple cell types in the microenvironment in numerous diseases and aging. In this issue of the *JCI*, Cao et al., using an elegant set of genetic mouse models and pharmacological approaches, demonstrated that gasdermin E (GSDME) was required in myeloid cells after sterile muscle injury to normally regenerate muscle and that downstream IL-18 release prevented intramuscular ectopic fat deposition. *GSDME* expression was reduced in human muscles from aged individuals, and *Gsdme* was increased after muscle injury in young, but not old, mice. The ability of IL-18 to partially improve regeneration in aged GSDME-knockout mice demonstrates the potential clinical relevance of this finding in dysregulated muscle regeneration associated with aging.

## Skeletal muscle regeneration

**Figure 1:**
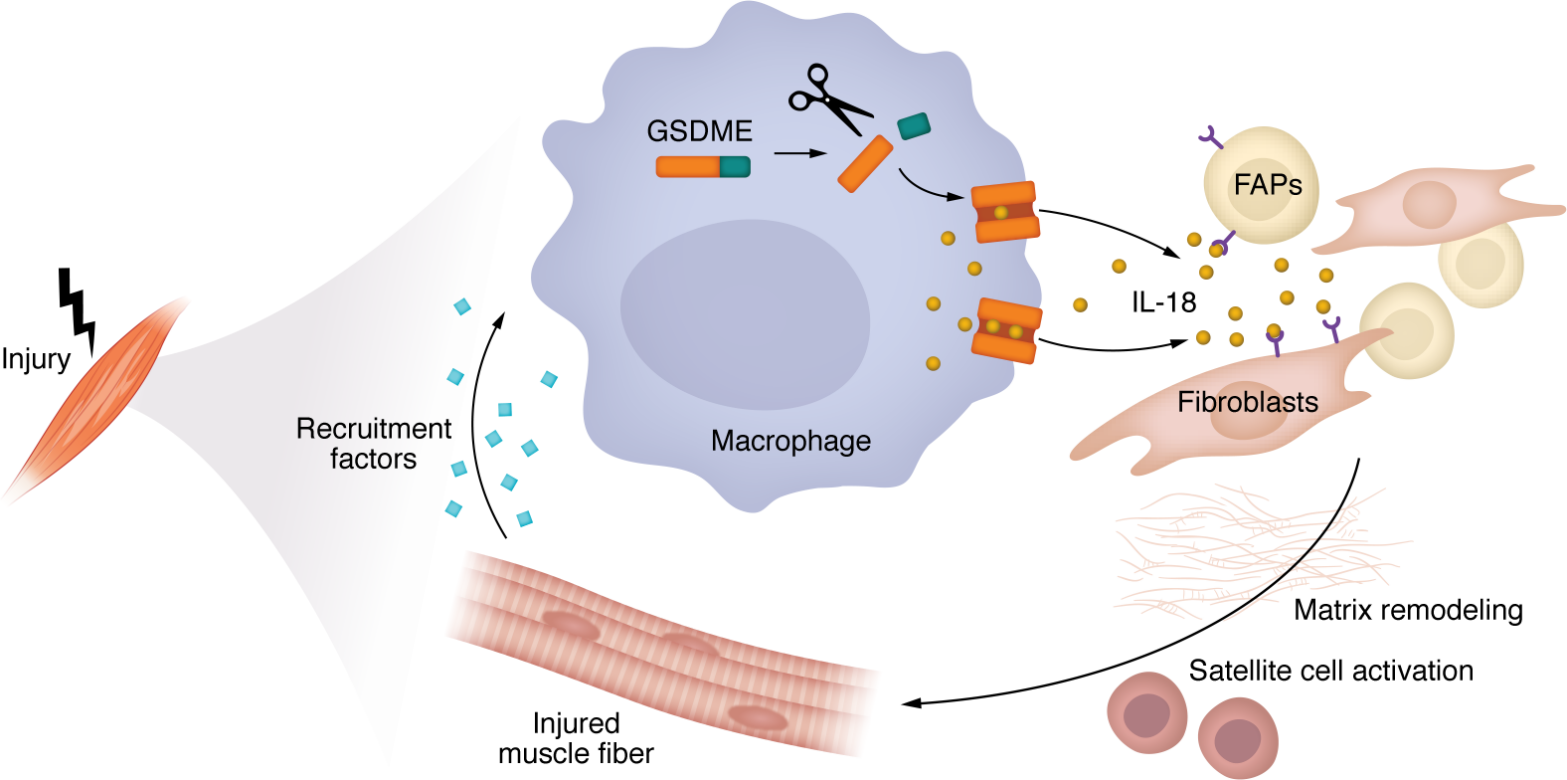
Myeloid cell GSDME expression coordinates skeletal muscle repair by facilitating IL-18 signaling to FAPs and fibroblasts. In injured muscle, crosstalk between multiple cell types is necessary for optimal skeletal muscle repair and regeneration. Macrophages recruited to the site of injury initiate the process of repair. Cao et al. (4) showed that GSDME in these macrophages causes pyroptosis and facilitates their release of IL-18, which maintains macrophage populations in the injured tissue, and signals to FAPs and fibroblasts to prevent maladaptive replacement of muscle fibers with fibrotic and fatty tissues. Delayed muscle regeneration and metabolic skewing toward adipogenesis and fat storage in Gsdme-deficient mice resemble age-related dysregulation of muscle repair in humans, underscoring the potential clinical relevance of reduced GSDME expression that Cao et al. observed in aged individuals.

Skeletal muscle weakness is a significant cause of morbidity and mortality across a spectrum of diseases and due to aging (1). In young individuals, skeletal muscle has the exceptional ability to entirely regenerate during a 2-week period, but this process becomes less seamless with disease and aging, leading to smaller muscle fibers and accumulation of either fat or fibrosis, weakening muscles (2). It is becoming increasingly clear that the skeletal muscle regenerative process is orchestrated by communication between the multiple cell types in the microenvironment including muscle fibers, muscle stem cells (MuSCs), immune cells, and fibro-adiopogenic progenitors (FAPs)/fibroblasts (Figure 1). This process can be easily studied in mouse models using severe chemical injury to an individual muscle using a single injection of either barium chloride or cardiotoxin. In contrast to skeletal muscle, the striated muscle of the heart has limited to no regenerative capacity. Thus, defining the mechanisms and intercellular communication underlying skeletal muscle regeneration could have an impact on development of therapeutic approaches for the repair of multiple tissues.

## Gasdermins

Gasdermin E (GSDME) belongs to the gasdermin superfamily (comprising GSDMA–GSDME) known to initiate pyroptosis, a form of programmed cell death that is followed by the release of inflammatory mediators through pores formed by the N-terminal fragments after proteolytic cleavage of gasdermins. GSDMD has been studied extensively, but less is known about functions of GSDME, particularly in muscle regeneration. Myeloid cell knockout of GSDMD in mice after cardiotoxin-induced muscle injury shows that GSDMD does not cause pyroptosis, but it leads to secretion of metabolites including the bioactive lipid 11,12-epoxyeicosatrienoic acid to contribute to muscle repair through crosstalk with MuSCs (3).

In this issue, Cao et al. addressed the role of GSDME in skeletal muscle regeneration using human samples, sophisticated mouse models, pharmacology, transcriptomics, mass spectrometry untargeted lipidomics, single-cell sequencing, flow cytometry, and pathology (4).

## GSDME regulates macrophage-to-FAP signaling in skeletal muscle regeneration

Building on their observation that *GSDME* gene expression was increased during muscle regeneration after injury in humans, the authors systematically assessed the muscle-specific effects of global *Gsdme* knockout in mice (4). In healthy muscle, following an acute sterile injury, myeloid immune cells peak at 2–3 days and initiate the process of repair and regeneration. FAPs and fibroblasts then peak 5–7 days after injury, contributing to matrix remodeling that supports satellite cell activation into MuSC proliferation and differentiation, leading to complete muscle regeneration within approximately 14 days after injury (5). In aging and chronic diseases such as muscular dystrophies, this well-coordinated process of muscle repair and regeneration becomes impaired, leading to the fatty and fibrotic replacement of muscle fibers.

In Cao et al.’s study (4), cardiotoxin injury in the global *Gsdme* knockouts resulted in delayed regeneration of muscle tissue and a switch from oxidative metabolism to lipid storage and adipogenesis, resulting in pathological fat accumulation. The role of GSDME in fat metabolism was further assessed by an elaborate experiment consisting of time-series transcriptomic analyses and an endpoint mass spectrometry untargeted lipidomic analysis after muscle injury comparing WT mice with the *Gsdme*-knockout mice. These analyses further confirmed that GSDME deficiency leads to enhanced myosteatosis.

The authors then began to determine molecular mechanisms downstream from GSDME in muscle regeneration. GSDME was expressed at the highest levels in mononuclear cells within the muscle regeneration microenvironment, identified as macrophages and FAPs. GSDME-initiated pyroptosis after muscle injury was confirmed by the canonical release of IL-18 and IL-1β from the N-terminal pores, including higher expression in CCR2^+^ monocyte–derived macrophages. Reexpression of GSDME in myeloid cells, but not FAPs from *Gsdme*-knockout mice, prevented myosteatosis and improved muscle function and all indicators of normal regeneration after injury. Neutralizing antibody treatment and single-cell transcriptomics were used to demonstrate that IL-18, but not IL-1β, release from monocyte-derived macrophages activates the transcriptional regulators KLF4 and JUN to maintain tissue-resident macrophages (TRMs) and prevent skewing of FAPs toward adipogenic states.

Since muscle regeneration is known to be impaired in aging, Cao et al. then investigated samples from young and aged patients. They showed reduced *GSDME* gene expression in muscles from aged patients (>70 years) undergoing abdominal surgery compared with young patients (<45 years). The cleaved forms of GSDME and IL-18 were also reduced in injured muscles from old compared with young mice. IL-18 administration partially improved the aging-related muscle regeneration abnormalities in old mice. However, IL-18 has been found to be increased in sarcopenic compared with nonsarcopenic aged individuals and decreased after interventions to improve muscle mass (6), suggesting that IL-18 may play different roles in muscle wasting and regeneration. Further investigations will be needed to thoroughly understand any potential benefit of IL-18 in specific clinical circumstances.

## Intercellular communication in skeletal muscle regeneration

Numerous studies now demonstrate that crosstalk between cell types in the damaged skeletal muscle environment regulates repair and regeneration (Figure 1). Although the myeloid populations in acute injury and chronic muscle diseases such as Duchenne muscular dystrophy mouse models are generally similar, their transcriptional signatures can differ (7, 8). The authors identified a macrophage cluster characterized by *Lyve1*, *Cd163*, and *Txnip*, which is suggestive of highly expanding TRMs, to be substantially reduced in the absence of GSDME. This TRM population was protective against myosteatosis and activated only when CCR2^+^ monocyte–derived macrophages were able to infiltrate regenerating muscles. Understanding TRMs and their therapeutic relevance in skeletal muscle and disease is an active area of research, given their roles in maintaining muscle homeostasis, repair, and regeneration (9). Of note, a recent study in a Duchenne muscular dystrophy mouse model identified the expansion and pathogenic role of TRMs in the absence of infiltrating macrophages in dystrophic skeletal muscles (10). These data suggest that signaling from macrophage populations may differ between normal muscle regeneration and disease, so therapeutic conclusions should only be inferred after first testing hypotheses in the most relevant disease model.

FAPs have also been demonstrated to support myogenesis, repair, and regeneration in acute injury and aging (11–14). In Cao et al.’s study, the subpopulation of *Pi16^+^Dpp4^+^* basal state FAPs were not altered by GSDME. However, *Dpp4*^+^ FAPs have been shown as the source of CSF1 for self-renewal of TIM4*^+^* TRMs in adult skeletal muscle (15). The higher proportion of the adipogenic FAP *Hsd11b1^+^Mme^+^Enpp2^+^* subpopulation and lower proportion of fibroblast-like *Fmod*^+^*Wif1*^+^*Comp*^+^ subpopulation that Cao et al. observed in *Gsdme* knockouts supports the view that alterations of different FAP subpopulations can carry out communication between different cell types that support muscle regeneration.

The contribution of immune cells to regeneration and their signaling to MuSCs is well documented (16). With the advent of single-cell technologies, specific macrophage subpopulations that signal to FAPs are being identified (17). Communication from FAPs/fibroblasts to MuSCs has also been demonstrated (13). Differentiated muscle cells (myotubes) have also been shown to secrete numerous cytokines and fibrotic factors that regulate FAP/fibroblast gene expression and function and are altered by disease (18). The current study by Cao et al. introduces GSDME as a player in the complex coordination of signaling in the skeletal muscle microenvironment. Further understanding of the crosstalk between cell types and the redundancy, overlap, and antagonism of various signals in health, aging, and different diseases that affect muscle will be required to effectively modulate efficiency of skeletal muscle regeneration.

## Conflict of interest

JARF’s spouse owns stock in Solid Biosciences.

## Version 1 04/15/2026

Electronic publication
